# A novel *PHKA2* variant in a Chinese boy with glycogen storage diseases type IXa

**DOI:** 10.3389/fendo.2023.1332450

**Published:** 2023-12-20

**Authors:** Hongdan Zhu, Tao Zhang, Hua Yuan, Yan Chen, Jinlong Ding, Haigang Ding, Xiaoliang Shi, Dalei Gu, Yingying Ma

**Affiliations:** ^1^ Shaoxing Maternity and Child Health Care Hospital, Shaoxing, Zhejiang, China; ^2^ Obstetrics and Gynecology Hospital of Shaoxing University, Shaoxing, Zhejiang, China; ^3^ Beilun District People’s Hospital, Ningbo, Zhejiang, China

**Keywords:** glycogen storage disease, X-chromosome recessive genetic diseases, PHKA2 gene, whole exome sequencing, 3D structure

## Abstract

**Background:**

Glycogen storage diseases (GSDs) are a group of heterogeneous inherited metabolic disorders with an incidence of 4%–5%. There are 19 types of GSDs, making diagnosis one of the greatest challenges.

**Methods:**

The proband and his parents were referred to our hospital for genetic diagnosis. Ultrasound screening suggested hepatomegaly. A novel insertion variant NM_000292 c.1155_1156insT (p. 386N>*) in *PHKA2* gene was identified using trio whole exome sequencing (Trio-WES), which resulted in the codon of amino acid 386 from asparagine to termination (p. 386N>*). The 3D mutant protein structure was predicted using AlphaFold, and the results showed that the truncated PHKA2 protein contained 385 of the 1,235 amino acids of the mature protein.

**Conclusion:**

We describe a previously unreported case of a GSDs IXa type Chinese boy caused by a novel *PHKA2* variant. This clinical case contributes to the understanding of the characteristics of GSDs type IXa and expands the variants spectrum of genes related to GSDs type IXa. Our findings demonstrated the significance of genetic testing in the diagnosis of GSDs.

## Introduction

Glycogen storage diseases (GSDs) are a group of heterogeneous genetic metabolic disorders caused by abnormal function of enzymes that control glycogen synthesis, regulation, and degradation (1, 2). GSDs can be divided into 19 types according to the specific enzyme deficiency and occupied tissues, among which are types I, VI, VIII, and IX. The primarily affect the liver and have a direct influence on blood glucose level (3, 4).

Type IXa GSD is mainly characterized by hepatomegaly and growth retardation in infancy. The clinical and biochemical abnormalities often improve or disappear with age. The severity of the disease is usually mild, and the majority of cases have a good prognosis. The incidence of one type of GSDs is not high, but the overall incidence is 4%–5%. Type IXa is considered to be the most common of all GSDs, with a prevalence of 1/100,000 (5). Thus, accurate detection of this rare genetic disease is an important strategy to improve the quality of newborns’ life.

Monogenic genetic disease refers to the genetic disease caused by single gene abnormality, also known as Mendelian genetic disease, which can be classified as autosomal dominant or recessive genetic disease, and X-chromosome dominant or recessive genetic disease. Type IX is caused by deficient activity of phosphorylase kinase (PHK), and its various subunits are encoded by different genes, including α (*PHKA1* and *PHKA2*), β (*PHKB*), ɣ (*PHKG1* and *PHKG2*), and δ (*CALM1, CALM2*, and *CALM3*). The gene encoding the α subunit of *PHK* is located on the X chromosome; therefore, GSD IXα has X-linked recessive inheritance characteristics, which mainly manifests in male individuals. Primary GSD type IXα (OMIM:306000)-associated *PHKA2* gene is characterized by hepatomegaly, growth retardation, elevated glutamic pyruvate aminotransferase and glutamic oxaloacetate aminotransferase, hypercholesterolemia, hypertriglyceridemia, and excessive fasting ketones. These clinical and biochemical abnormalities gradually disappear with age. Most of the adult patients are asymptomatic.

In this study, we reported a 2-year-old Chinese boy with hepatomegaly detected by ultrasound screening. Whole exome sequencing (WES) revealed a novel variant, NM_000292 c.1155_1156insT (p. 386N>*) in *PHKA2*, which was an insertion variant, caused by a nucleotide A insertion between 1155 and 1156 position, resulting in the asparagine codon at position Asp being changed to a stop codon. The patient’s mother was found to carry the heterozygous variant, NM_000292 c.1155_1156insT (p. 386N>*) in *PHKA2*, indicating that the patient inherited the variant from his mother. His mother carried the variant NM_000292 c.1155_1156insT (p. *) in *PHKA2* gene, suggesting that the child inherited it from his mother. The patient was evaluated by a dietitian and recommended a diet high in protein and complex carbohydrates. However, the patient was unable to follow a strict diet and eventually received a liver transplant. Our findings demonstrated the significance of genetic testing in the diagnosis of GSD type IXα. We hope that this work will contribute to the interpretation of *PHKA2* variants by geneticists and help improve the treatment of patients with GSD.

## Materials and methods

### Sample collection

A 2-year-old Chinese boy, who presented hepatomegaly by ultrasound screening, was enrolled in the Shaoxing Maternity and Child Health Care Hospital (Shaoxing, Zhejiang Province, China). The venous blood samples of the proband and his parents were collected for this study. The study was approved by the institutional ethics committee of Shaoxing Maternity and Child Health Care Hospital. The family members had signed informed consent documents for participation and publication.

### Trio whole exome sequencing

Genomic DNA was extracted from venous blood of the proband and his parents using a DNEasy Blood and Tissue Kit (Qiagen, Hilden, Germany) according to the manufacturer’s procedures. For trio WES (proband and his parents), the genomic DNAs were enriched for coding exons using Agilent SureSelect Low Input Reagent Kit and sequenced on Illumina HiSeq X Ten platform. The proband sequencing data captured 99.89% of coding regions across 58,682,415 bp length of 25,701 genes in total. The average sequence depth is ×263.37 and 98.75% of targeted regions with average depth > ×20.

### Data analysis

The AfterQC (6) was used to evaluate the sequencing quality of the original sequencing data, and the low-quality and contaminated reads were removed. To detect the potential variants in the family, we performed bioinformatics processing and data analysis after receiving the primary sequencing data. We used previously published filtering criteria to generate “clean reads” for further analysis (7). After data were aligned to human reference hg19 by BWA software as described previously (8), the single nucleotide variants (SNVs) and indels in genome were called by using the GATK software (9). Then, we used the 1000 Genomes database (1000 human genome dataset), Genome AD (Genome Aggregation Database dataset) 2.1.1, and ExAC (The Exome Aggregation Consortium dataset) to screen the SNV and indels, and used the OMIM, HGMD, and Clinvar databases to filter the reported variants. dbNSFP database was used to predict the pathogenicity of missense and splice variants. All variants sites were classified according to ACMG genetic variation classification criteria and guidelines. Finally, Sanger sequencing method was used to verify all possible pathogenic variants.

### Protein structure prediction

The protein sequence with 1,235 amino acid residues of PHKA2 was downloaded from NCBI (NP_000283.1). The wild- and mutant-type 3D structure of the PHKA2 protein was predicted using AlphaFold web server (https://alphafold.ebi.ac.uk/) (9). The best model was selected based on I-TASSER prediction (10). The final predicted structure was visualized using PyMOL program (https://pymol.org/).

## Results

### Clinical presentation

**Figure 1:**
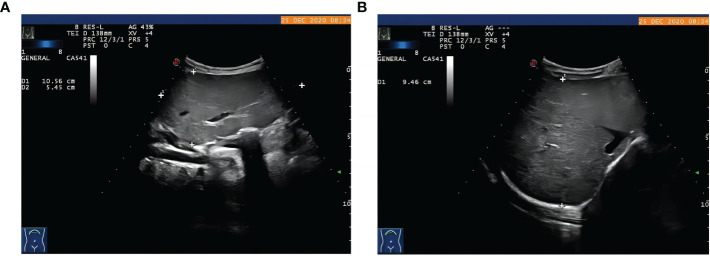
The proband with hepatomegaly. Abdominal ultrasound examination showed hepatomegaly with **(A)** the thickness of left liver lobe of 5.45 cm, vertical diameter of 10.56 cm, **(B)** right liver oblique diameter of 9.46 cm, normal echotexture, and no splenomegaly.

**Figure 2:**
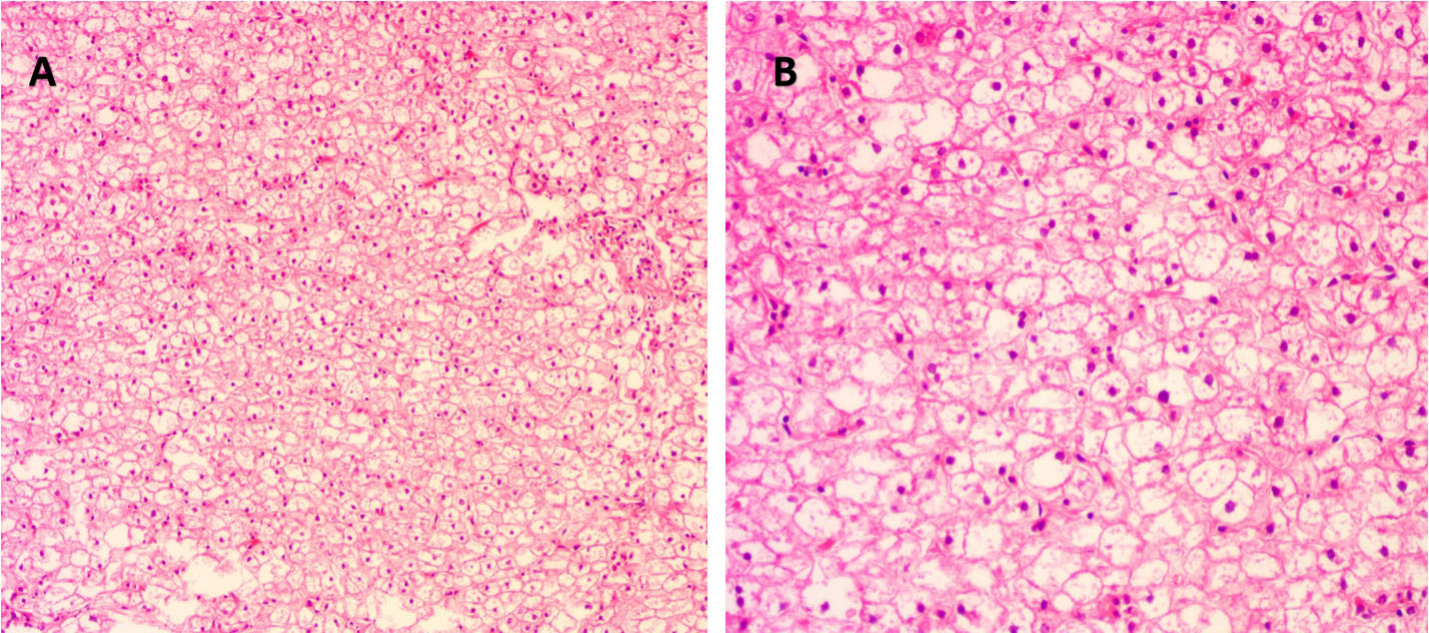
Histological examination of liver tissue of the proband. **(A)** Under magnification ×100 and **(B)** under magnification ×200. Hematoxylin and eosin (H&E) staining showed degenerated and swollen hepatocytes with bright cytoplasm and small nuclei.

The proband was the second child of a healthy Chinese non-consanguineous parents, born by vaginal delivery with a birth weight of 2,600 g, 17 days ahead of the expected date of delivery. At 2 years old, he was referred to a local hospital due to distended abdomen. Abdominal ultrasound examination indicated hepatomegaly with a left liver lobe thickness of 5.45 cm, vertical diameter of 10.56 cm, and right liver oblique diameter of 9.46 cm, with normal echotexture and no splenomegaly (**Figure 1**). He received blood biochemical analysis (**Table 1**). Subsequently, the proband and his parents were referred to our hospital for genetic diagnosis. The proband was developmentally delayed with a height of 81 cm and weight of 11 kg. Combined with the clinical characteristics and blood biochemical analysis results, it was inferred that the patient might have GSDs. The patient was advised to have a diet by high protein and high complex carbohydrates. However, the patient could not endure strict dietary control and eventually underwent liver transplantation. The following histological examination of the post-operative pathological liver section was performed, and hematoxylin–eosin (H&E) staining showed that the hepatocytes were degenerated and swelling, with bright cytoplasm, and small nuclei (**Figure 2**).

**Table 1:** Blood biochemical analysis of the proband.

Content	Abbreviation	Value
Alanine transferase	ALT	657 U/L
Aspartate transaminase	AST	658 U/L
Glutamine transpeptidase	GGT	116 U/L
Glucose	GLU	3.01 mmol/L
Triglyceride	TG	2.60 mmol/L
Lactate dehydrogenase	LDH	495 μ/L
Immunoglobulin G	IGG	6.27 g/L
Immunoglobulin A	IGA	0.64 g/L
α-L-fucosidase	AFU	64.4 U/L
Lactic acid	LA	2.9 mmol/L

### Identification of a novel variant in *PHKA2* gene

**Figure 3:**
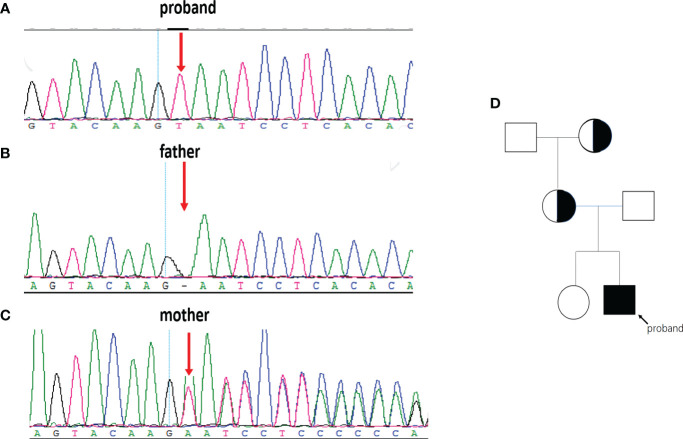
The *PHKA2* variant in the family. WES and Sanger sequencing confirmed the presence of the NM_000292 c.1155_1156insT (p. 386N>*) in *PHKA2* gene of the proband **(A)**. The father **(B)** carried a normal allele. The mother **(C)** was heterozygote carrier. Pedigree of the family **(D)** showed that the mother and the grandmother were heterozygote carriers. The proband was indicated by the arrow.

We performed WES on the proband and identified a novel variant, NM_000292 c.1155_1156insT (p. 386N>*) in the *PHKA2* gene, which is an insertion mutation caused by the insertion of nucleotide A between position 1155 and 1156, resulting in the asparagine codon being changed to a stop codon (**Figure 3A**). After a trio analysis, it was found that the mother carried a heterozygous variant, NM_000292 c.1155_1156insT (p. 386N>*), in the *PHKA2* gene, indicating that the patient inherited the mutation from his mother (**Figures 3B, C**). Subsequently, Sanger sequencing was performed to verify this insertion mutation in all family members, and the results showed that it was a recessive genetic disease on the X-chromosome (**Figure 3D**). According to the American College of Medical Genetics and Genomics (ACMG) guidelines (11), the NM_000292, c.1155_1156insT (p. 386N>*) in *PHKA2* was predicted to be pathogenic variants because of the evidence chain (PVS1+PM2+PP3). The sequence data had been submitted to GenBank with the accession number 2705096.

### Effect of mutations on protein structure

**Figure 4:**
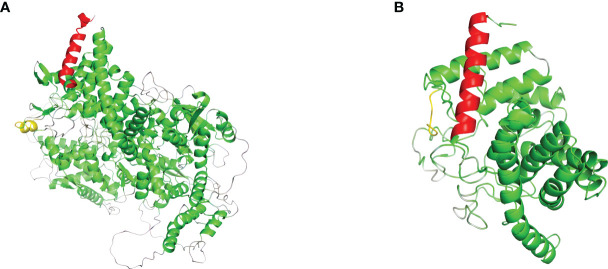
Effects of *PHKA2* variants on the protein structure. **(A)** The wide-type structure of the PHKA2 protein predicted by AlphaFold. The red part shows the N-terminal region, and the yellow part represents as amino acid residues at position 386. The mutant protein structure shown in **(B)** contained a truncated PHKA2 protein that consist 385 of the 1,235 amino acids of the mature protein.

In order to analyze the effect of the novel variant, NM_000292 c.1155_1156insT (p. 386N>*) on protein structures of PHKA2. We used AlphaFold web server (https://alphafold.ebi.ac.uk/) (9) to predict changes in PHKA2 protein structures when there were mutations in the sequence. The NM_000292 c.1155_1156insT (p. 386N>*) variant in *PHKA2* gene will change the 3D structure to a truncated PHKA2 protein that consist 385 of the 1,235 amino acids of the mature protein (**Figure 4**). The structural change would alter the conformation of the PHKA2 protein and affect the protein stability and binding facility.

## Discussion

Most GSDs are referred to hospitals for developmental delay, hepatomegaly, and other abnormalities. However, it is still challenging to identify 19 different types of GSDs based on clinical findings. Compared with other types of GSDs, type IXa generally has mild symptoms, and many patients have no symptoms or only mildly elevated blood triglyceride levels. Liver biopsy and staining revealed swollen hepatocytes due to glycogen accumulation in the cytoplasm.

In addition, no fibrosis or steatosis was observed in the liver, suggesting a mild GSD in the proband. Hepatomegaly and elevated serum liver transaminases are consistent with the most common characteristics of GSDs. Next-generation sequencing has been recommended by the ACMG as a useful method for diagnosing different types of GSD (12). The application and promotion of high-throughput sequencing technology has improved the diagnostic yield of GSDs, including many novel variants (12).

The *PHKA2* gene is located on the long arm of the X chromosome (Xp22.2-22.1) and encodes a α subunit consisting of 33 exons. Up to now, more than 130 *PHKA2* gene variants of GSD type IXa has been reported. These variants include small deletions, gross deletions variants, small insertions, missense, and splicing (13, 14). There was heterogeneity among subjects, even among individuals carrying the same *PHKA2* variant. Almost all the previously published cases were male, but several female cases have also been found due to X chromosome inactivation (15). In this study, we identified a novel insertion variant NM_000292 c.1155_1156insT (p. 386N>*) in *PHKA2* gene, which led to an accurate diagnosis of the proband as GSD type IXa. The insertion variant c.1155_1156insT changed the asparagine (N) to termination (*) at codon position 386, resulting in a truncated PHKA2 protein containing 385 of the 1235 amino acids as generated. The truncated PHKA2 was broken in the GH-15 like domain, resulting in a lack of the CBL-like domain and the entire C-terminal region of PHKA2. The proband inherited the variant from his mother, and the mother inherited the same variant from the proband’s grandmother. Both the female variant carriers showed normal liver functions.

**Figure 5:**
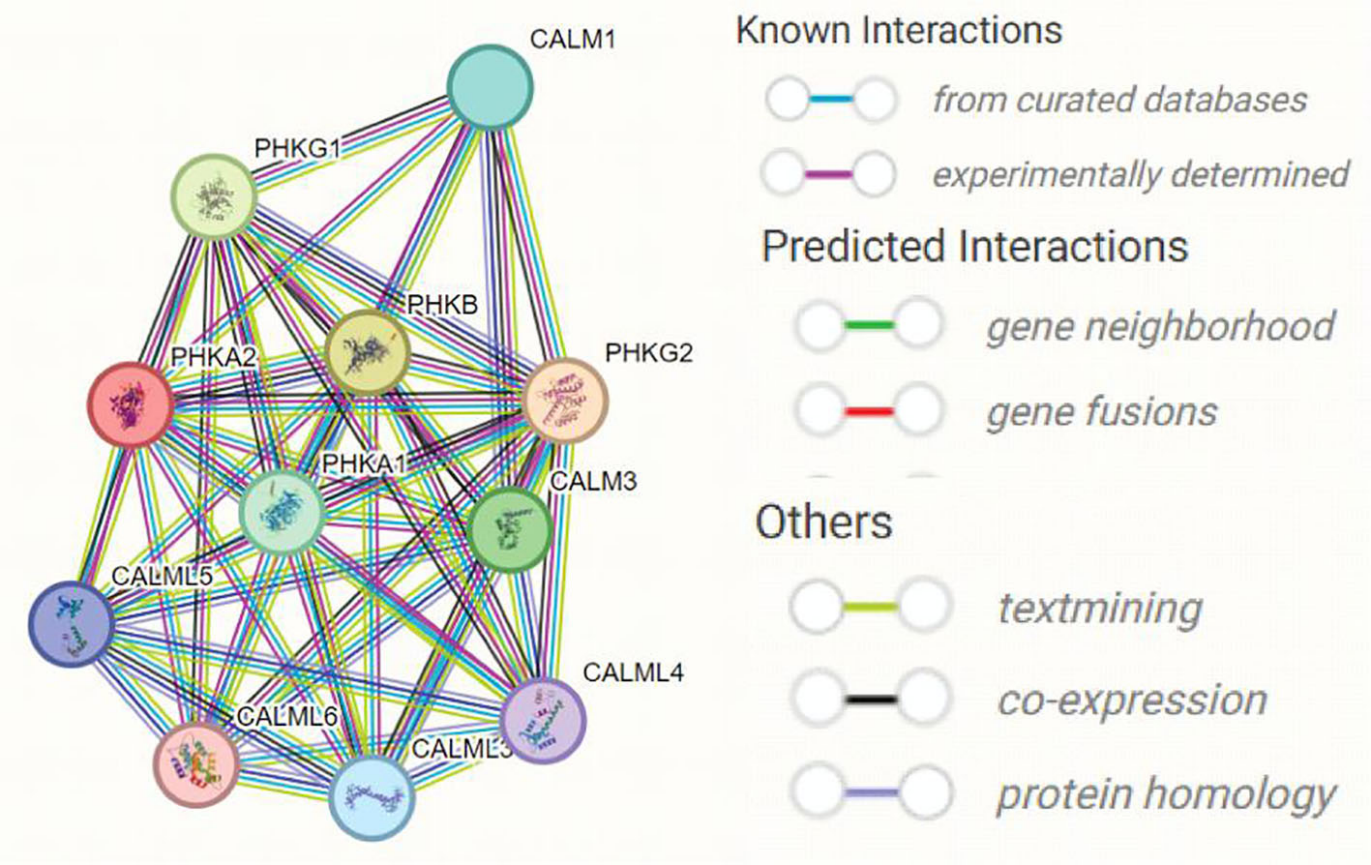
The predicted interacting proteins of PHKA2 using STRING website.

Multiple subunits of phosphorylase kinase (PHK) are encoded by different genes, including α (PHKA1 and PHKA2), β (PHKB), γ (PHKG1 and PHKG2), and δ (CALM1, CALM2, and CALM3). We used STING software to predict the interacting proteins with PHKA2 and found several catalytic chain proteins of phosphorylase b kinases, including PHKA1, PHKG1, PHKG2, and PHKB, which regulate glycogenolysis by activating glycogen phosphorylase through phosphorylation (**Figure 5**). CALM1, CALM3, and four calmodulin-like proteins including CALML3, CALML4, CALML5, and CALML6 were also predicted to interact with PHKA2 (**Table 2**). Normal keratinocytes suddenly and transiently increase calmodulin-like proteins during growth, and these calmodulin-like proteins may interact with PHKA2 protein to play an important role in the control of glycogen synthesis, regulation, and degradation.

**Table 2:** Names and functions of predicted PHKA2-interacting proteins.

Interacting proteins	Function of proteins
PHKA1	Phosphorylase b kinase regulatory subunit alpha, skeletal muscle isoform; phosphorylase b kinase catalyzes the phosphorylation of serine in certain substrates
PHKG1	Phosphorylase b kinase γ catalytic chain, skeletal muscle/heart isoform; catalytic subunit of the phosphorylase b kinase (PHK), which mediates the neural and hormonal regulation of glycogen breakdown (glycogenolysis) by phosphorylating and thereby activating glycogen phosphorylase.
PHKG2	Phosphorylase b kinase γ catalytic chain, liver/testis isoform; Catalytic subunit of the phosphorylase b kinase (PHK), which mediates the neural and hormonal regulation of glycogen breakdown (glycogenolysis) by phosphorylating and thereby activating glycogen phosphorylase.
PHKB	Phosphorylase b kinase regulatory subunit β; phosphorylase b kinase catalyzes the phosphorylation of serine in certain substrates, including troponin I. The beta chain acts as a regulatory unit and modulates the activity of the holoenzyme in response to phosphorylation.
CLAM3	Calmodulin-3; calmodulin mediates the control of a large number of enzymes, ion channels, aquaporins, and other proteins through calcium binding. Among the enzymes to be stimulated by the calmodulin–calcium complex are a number of protein kinases and phosphatases.
CLAM1	Calmodulin-1 belongs to the calmodulin family.
CALML3	Calmodulin-like protein 3; may function as a specific light chain of unconventional myosin-10 (MYO10), also enhances MYO10 translation, possibly by acting as a chaperone for the emerging MYO10 heavy chain protein.
CALML4	Calmodulin-like 4 belongs to the calmodulin family
CALML5	Calmodulin-like protein 5 may be involved in terminal differentiation of keratinocytes
CALML6	Calmodulin-like 6 belongs to the calmodulin family

Prevention of rare genetic diseases is an important strategy to ensure the quality of the birth population. However, a variety of recessive disease-causing genes are present in every healthy person. Carriers of recessive genetic diseases are phenotypically normal, routine prenatal testing often fails to detect newborns, and the pathogenic variants are passed from generation to generation until the carriers have children (16, 17). Therefore, it is necessary to screen the couples of recessive gene carriers to understand and assess their potential risk of having children. If the couple is at risk of having a child with a rare disease, a healthy child can be born through assisted reproductive technology using preimplantation genetic testing (PGT) (18). In this study, a 2-year-old boy was found to have hepatomegaly and developmental delay. A novel variant in *PHKA2* gene was detected by WES. Therefore, he was diagnosed as having GSD type IXa. The proband was evaluated by a dietitian and advised to have a diet high in protein and complex carbohydrates. However, he was unable to follow a strict diet and eventually underwent a liver transplant. Newborn screening based on next-generation sequencing technology can detect pathogenic genetic variants as early as possible at birth, and timely intervention treatment measures can be carried out to improve the life of newborns.

In conclusion, the use of high-throughput sequencing technology has provided insights into the cause of this proband’s disease. The novel insertion variant NM_000292 c.1155_1156insT (p. 386N>*) in *PHKA2* gene might be the cause of GSD type IXa. This mutation represented a novel mutation, as it had not been previously reported. However, the exact effect mechanism of this mutation to PHKA2 function requires further study. This study showed that clinical characteristics combined with WES can provide accurate molecular diagnosis, and patients will benefit from this molecular diagnosis.

## Data availability statement

The datasets presented in this study can be found in online repositories. The names of the repository/repositories and accession number(s) can be found below: https://www.ncbi.nlm.nih.gov/genbank/, 2705096.

## Ethics statement

The study was approved by the institutional ethics committee of Shaoxing Maternity and Child Health Care Hospital. The studies were conducted in accordance with the local legislation and institutional requirements. Written informed consent for participation in this study was provided by the participants’ legal guardians/next of kin. Written informed consent was obtained from the individual(s), and minor(s)’ legal guardian/next of kin, for the publication of any potentially identifiable images or data included in this article.

## Author contributions

HZ: Funding acquisition, Project administration, Writing – original draft. TZ: Conceptualization, Data curation, Funding acquisition, Writing – original draft. HY: Formal Analysis, Investigation, Writing – review & editing. YC: Resources, Software, Writing – original draft. JD: Data curation, Writing – original draft. HD: Investigation, Methodology, Software, Writing – review & editing. XS: Formal Analysis, Methodology, Project administration, Writing – review & editing. DG: Conceptualization, Investigation, Resources, Visualization, Writing – original draft. YM: Conceptualization, Writing – original draft, Writing – review & editing.
